# A temperature-responsive PLA-based nanosponge as a novel nanoadjuvant and efficient delivery carrier of Ag85B for effective vaccine against *Mycobacterium tuberculosis*

**DOI:** 10.1186/s12964-025-02105-2

**Published:** 2025-04-01

**Authors:** Jin-Seung Yun, Soo-Min Kim, Jin Sil Lee, Su Hyun Jeong, Hyeryeon Oh, Panmo Son, Sunghyun Kim, Young-Ran Lee, Eunkyung Shin, Sang-Jun Ha, Yong-Woo Jung, Dokeun Kim, Hye-Sook Jeong, Won Il Choi

**Affiliations:** 1 National Institute of Infectious Disease, Korea National Institute of Health, 212, Osongsaengmyeong 2-ro, Osong-eup, Heungdeok-gu, Cheongju, Chungbuk 28160 Republic of Korea; 2 Department of Biochemistry, College of Life Science and Biotechnology, Yonsei University, Seoul, 03722 Republic of Korea; 3 College of Pharmacy, Korea University, Sejong, 30019 Republic of Korea; 4 Bio-Convergence Materials R&D Division, Korea Institute of Ceramic Engineering and Technology, 202, Osongsaengmyeong 1-ro, Osong-eup, Heungdeok-gu, Cheongju, Chungbuk 28160 Republic of Korea; 5 Drug Manufacturing Center, Daegu-Gyeongbuk Medical Innovation Foundation (K-MEDI Hub), Daegu, 41061 Republic of Korea; 6 Department of Bioengineering, Hanyang University, Seoul, 04763 Republic of Korea; 7 School of Materials Science and Engineering, Department of Nanobio Materials and Electronics, Gwangju Institute of Science and Technology, 261 Cheomdan-gwagiro, Buk-gu, Gwangju, 500-712 Republic of Korea; 8 Department of Applied Bioengineering, Graduate School of Convergence Science and Technology, Seoul National University, Seoul, 08826 Republic of Korea

**Keywords:** PLA, Nanosponge, Nanoadjuvant, New TB vaccine, BCG-booster vaccine, Tuberculosis

## Abstract

**Background:**

Tuberculosis (TB) is a contagious disease and the second leading cause of death worldwide. The Bacille Calmette–Guérin (BCG) vaccine, the only licensed TB vaccine, has insufficient protective efficacy in adults, necessitating the development of new TB vaccines. Ag85B, a protein-subunit TB vaccine, is a promising candidate due to its high immunogenicity. However, its hydrophobicity presents challenges in manufacturing, expression, and purification, and Ag85B alone does not elicit sufficient immune stimulation. To overcome these limitations, this study aimed to design a temperature-responsive amine-terminated polylactic acid (PLA)-based nanosponge (aPNS) as both a nanoadjuvant and an efficient delivery carrier for Ag85B.

**Methods:**

Ag85B was produced using an EZtag fusion tag vector, achieving high product yield and purity. It was then loaded into aPNS, a nanoparticle system with a PLA core and Pluronic shell, through a temperature-responsive process at 4 °C that preserved protein bioactivity. The stability and sustained-release profile of Ag85B@aPNS were evaluated. In vitro cytotoxicity and cellular uptake studies were conducted using macrophages. Protective efficacy and immunogenicity were assessed in *M. tuberculosis*-challenged mice and BCG-primed mice.

**Results:**

The Ag85B protein was successfully produced and loaded into aPNS, which exhibited good colloidal stability and a sustained-release profile. Neither the synthesized Ag85B nor the aPNS showed significant cytotoxicity. aPNS enhanced the cellular uptake of antigens by macrophages. Compared to BCG, Ag85B@aPNS demonstrated superior protective efficacy against *M. tuberculosis* in mice and improved immunogenicity in BCG-primed mice.

**Conclusion:**

Ag85B@aPNS is a viable candidate for TB vaccination, showing potential as both a standalone vaccine and a BCG-booster. Its ability to enhance immunogenicity and provide protection highlights its promise in addressing the limitations of current TB vaccines.

**Supplementary Information:**

The online version contains supplementary material available at 10.1186/s12964-025-02105-2.

## Plain language summary

Tuberculosis (TB) remains a major global health challenge, and while the current BCG vaccine provides some protection, its effectiveness varies and it does not fully prevent the disease in adults. To address this, researchers are developing new vaccines and boosters that enhance the immune system’s ability to fight TB. This study introduces a novel vaccine candidate called Ag85B@aPNS, which combines a TB-specific protein (Ag85B) with a specialized nanoparticle delivery system (aPNS). The researchers designed the aPNS to carry and protect the Ag85B protein, ensuring it remains stable and active for a longer time. These nanoparticles also respond to temperature changes, making them more effective in releasing the protein under human body conditions. Experiments showed that Ag85B@aPNS was safe for cells and enhanced the ability of immune cells to recognize and process the protein. This means the vaccine can better stimulate the immune system without causing harm. In animal studies, the vaccine was tested in mice infected with TB bacteria. Mice that received Ag85B@aPNS had fewer bacteria in their lungs and less lung damage compared to those given the standard BCG vaccine. Furthermore, when used as a booster in BCG-primed mice, the vaccine strengthened the immune response significantly. These findings suggest that Ag85B@aPNS is a promising candidate for improving TB vaccination strategies, either as a replacement for BCG or as a booster to enhance its effects. This could lead to better protection against TB in the future.

**Supplementary Information:**

The online version contains supplementary material available at 10.1186/s12964-025-02105-2.

## Introduction

Tuberculosis (TB), induced by *Mycobacterium tuberculosis*, is a contagious disease and the second leading cause of death worldwide [1–3]. In the past few years, global healthcare efforts have primarily focused on COVID-19, resulting in the deprioritization of strategies for eradicating TB [4]. According to the World Health Organization, in 2023, 7.5 million people were diagnosed with TB, and 1.3 million people died of TB [3, 5]. Because the severity of TB is aggravated with increased occurrence and mortality annually, a new breakthrough for diagnosis and appropriate prevention is urgently required [5, 6]. Bacille Calmette–Guérin (BCG)—an attenuated form of *Mycobacterium bovis*—is the only licensed vaccine against TB, and the protection BCG offers varies [7, 8]. The BCG vaccine frequently fails to provide adequate protective efficacy in adults and thus is a potential factor contributing to TB transmission in adults and adolescents [8, 9]. Current efforts to develop new TB vaccines are aimed at addressing this challenge.

Vaccine platforms, including inactivated subunits and DNA vaccines, are being extensively investigated. Inactivated and subunit TB vaccines, such as MTBVAC and M72/AS01, are considered the most promising candidates in clinical trials [10]. Subunit vaccines, particularly protein-subunit vaccines, offer the highest safety during manufacturing and use [11]. For example, the Ag85 complex, which comprises the abundantly secreted antigens Ag85A, Ag85B, and Ag85C, is considered a promising candidate for producing TB vaccines. Ag85 is involved in cell wall biosynthesis as a mycolyl transferase [12, 13]. In cases of TB infection, Ag85 activates the production of IFN-γ, stimulates the generation of Th1 lymphocytes, and induces cytotoxic lymphocytes, resulting in protective immunogenicity [14, 15]. Ag85B in the Ag85 complex is a highly immunodominant antigen with T-cell epitopes and has been detected in specific cell-mediated and humoral immune responses in infected patients [16]. However, unlike the process of manufacturing soluble proteins, manufacturing Ag85B is challenging owing to its hydrophobic characteristics of Ag85B [14, 17]. Therefore, innovative strategies are required to optimize the manufacturing process of Ag85B.

Despite its immunodominant properties, Ag85B alone does not induce an adequate protective immune response because the number of antigens is limited. Therefore, appropriate adjuvants such as immunostimulatory or delivery systems are necessary to elicit T cells and local immunity [18]. Previous studies have demonstrated that adjuvants play a significant role in effective TB treatment [11, 19]. Numerous nanomaterials are currently being explored as delivery systems capable of protecting antigens from degradation post-immunization [18]. Nanoparticles composed of polylactic acid (PLA), poly(lactic-co-glycolic acid) (PLGA), hyaluronic acid, chitosan, and cellulose are particularly favored due to their ability to prevent antigen degradation, ensure sustained antigen release, and facilitate intracellular delivery of antigens to antigen-presenting cells, depending on the administration site. These nanoparticles also exhibit advantageous physicochemical properties and surface chemistry [20–24]. Balleter et al. [25] developed a synthetic vaccine delivery platform by conjugating Ag85B to Pluronic-stabilized polypropylene sulfide nanoparticles via a reducible disulfide bond (NP-Ag85B) to enhance protection against TB. NP-Ag85B was administered to mice alongside the immunostimulatory oligonucleotide CpG. In dendritic cells, NP-Ag85B activated the inflammasome and increased IL-6 production in the presence of the Th1-promoting TLR9 ligand CpG. Vaccinated mice exhibited Th17 responses and enhanced polyfunctionality of Th1 responses. Malik et al. [26] evaluated a bivalent fusion antigen composed of ESAT6 and Ag85B protein (H1) encapsulated in PLGA NPs (H1 NP) using a water-oil-water solvent evaporation method. The nanoparticles exhibited a nanodiameter range of 200–280 nm, a negative surface charge, and over 85% encapsulation efficiency. In H1 NP-immunized mice, both humoral and cell-mediated immune responses were enhanced. Additionally, the mice demonstrated improved protection efficacy, as evidenced by a significant reduction in bacterial load in the lungs and spleen. Based on these reports, to enhance the immune response of the antigen, it is necessary to encapsulate the antigen into nanoparticles with additional immunostimulatory effects or to combine various antigens through fused conjugates. Consequently, there is a need for a novel delivery carrier that can sufficiently stimulate the efficacy of antigens even without supplementary immunostimulatory components or the use of fused antigens.

**Fig. 1:**
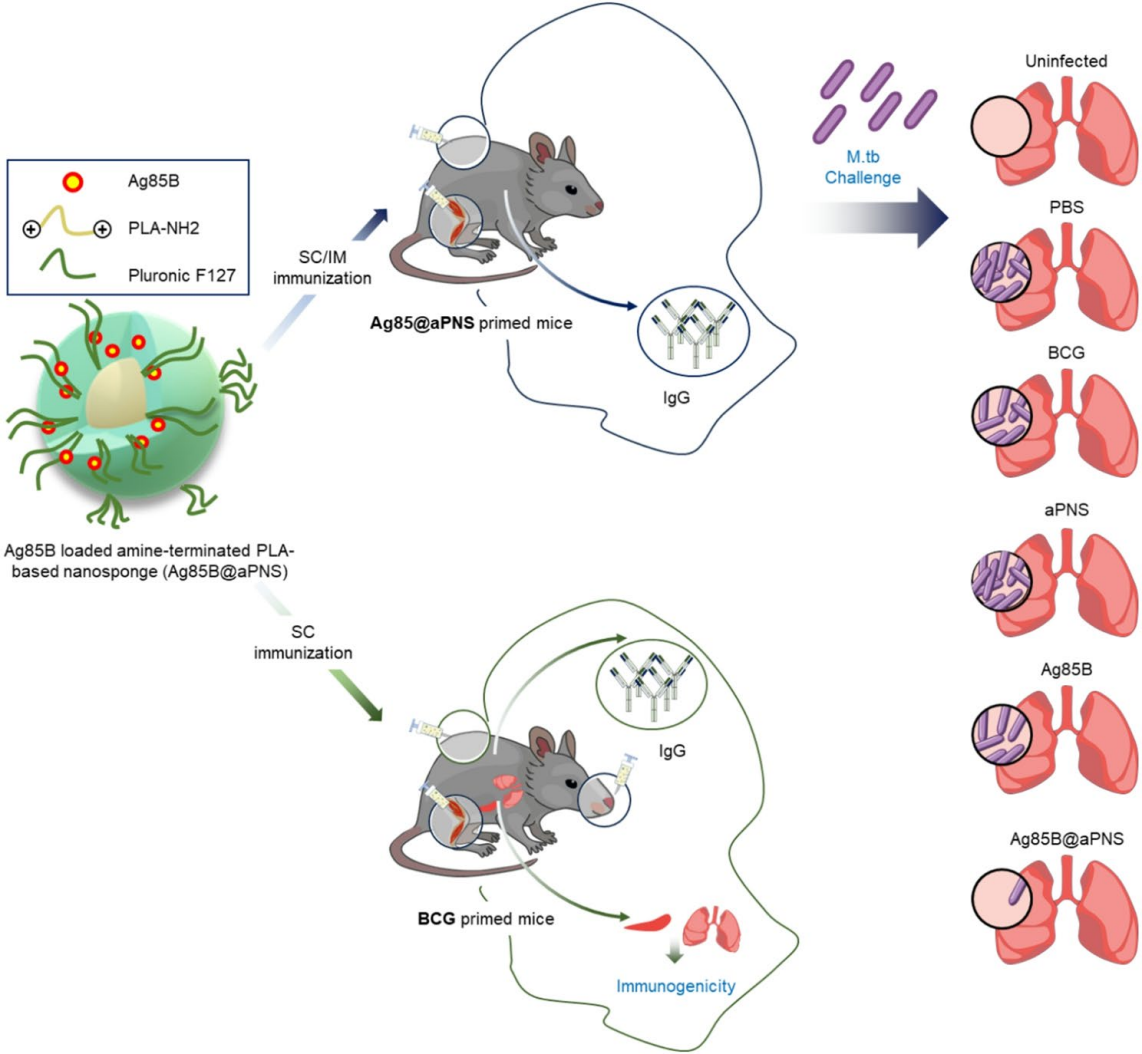
Schematic representation of the in vivo application of Ag85B@aPNS as a novel TB vaccine and BCG-booster vaccine for TB treatment

In this study, we developed a novel PLA-based nano-vaccine, temperature-responsive amine-terminated PLA-based nanosponge (aPNS). The aPNS consists of a PLA core and a Pluronic shell, serving as both a nanoadjuvant and an efficient delivery carrier for Ag85B. This system was fabricated through a simple nanoprecipitation method without the need for covalent conjugation, aiming to achieve effective TB vaccination (Fig. 1). In addition, Ag85B was successfully produced with a high product yield and purity using an EZtag fusion tag vector. The purified Ag85B was efficiently loaded into aPNS with volume expansion behavior at 4 °C in the absence of organic solvents, which helped preserve protein bioactivity. The Ag85B-loaded aPNS (Ag85B@aPNS) showed good colloidal stability after lyophilization, even without the addition of cryoprotectants; moreover, the colloidal stability was maintained for four weeks in a physiological environment. A sustained Ag85B release profile from aPNS was observed for 21 days. In addition, neither Ag85B nor aPNS showed notable cytotoxicity in macrophages, and aPNS improved the cellular uptake of the antigen. Moreover, Ag85B@aPNS stimulated better Th1 immune responses and reduced the lung load and inflamed areas in *M. tuberculosis-infected* mice. Furthermore, Ag85B@aPNS increased humoral and cell-mediated immune responses in BCG-primed mice. In summary, our results demonstrate that Ag85B@aPNS enhances the protective efficacy against *M. tuberculosis* challenges compared to BCG immunization and boosts the immunogenic effects of the BCG vaccine in BCG-primed mice. Therefore, we conclude that Ag85B@aPNS can serve as an effective BCG booster vaccine.

## Materials and methods

### Materials

*Escherichia coli* strain BL21(DE3) was purchased from Enzynomics (Yuseong-gu, Daejeon, Republic of Korea). Terrific Broth was purchased from Invitrogen (Waltham, Massachusetts, USA). Phenylmethanesulfonyl fluoride (PMSF) and ampicillin were purchased from Sigma-Aldrich (Burlington, Massachusetts, USA). Isopropyl β-D-1-thiogalactopyranoside (IPTG) was purchased from LPS Solution (Daedeok-gu, Daejeon, Republic of Korea). MonoQ and desalting columns were purchased from Cytiva (Amersham, England, UK). Tobacco etch virus (TEV) protease was purchased from GenScript (Piscataway, NJ, USA). Pierce high-capacity endotoxin removal spin columns were purchased from Thermo Fisher Scientific (Waltham, Massachusetts, USA). The EZtag vector was purchased from ToolBio (Yuseong-gu, Daejeon, Republic of Korea), and the pBT7-N-His vector was purchased from Bioneer (Daedeok-gu, Daejeon, Republic of Korea).

Ovalbumin (OVA), Pluronic F127 (PF127), and acetone were purchased from Sigma-Aldrich (St. Louis, MO, USA). Amine-terminated PLA was obtained from Polysciences, Inc. (Warrington, PA, USA). Hypure water (deionized water, DIW) and phosphate-buffered saline (PBS) solution (×1) were obtained from Hyclone (Logan, UT, USA). The bicinchoninic acid (BCA) protein assay kit was purchased from TaKaRa (Ohtsu, Shiga, Japan). RAW 264.7 macrophage were obtained from ATCC (TIB-71, Manassas, VA, USA). RPMI-1640 medium, fetal bovine serum (FBS), penicillin/streptomycin (P/S), and Dulbecco’s phosphate-buffered saline (DPBS) were obtained from Gibco (Waltham, MA, USA). The 3-(4,5-dimethilthiazol-2-yl)-2,5-diphenyltetrazolium bromide (MTT) assay kit was purchased from Abcam (Cambridge, Cambridgeshire, UK). Albumin-fluorescein isothiocyanate conjugate (OVA-FITC) was purchased from Sigma-Aldrich (St. Louis, MO, USA). All substrates and ELISpot kits were purchased from BD Biosciences (San Diego, CA).

### Preparation of Ag85B protein

Ag85B was synthesized by Bioneer (Daejeon, Republic of Korea) in a pBT7-N-His vector and subsequently cloned into the EZtag vector. His-Ag85B and EZtag-Ag85B plasmids were transformed into *E. coli* strain BL21(DE3). IPTG (1 mM) was then added, and the cells were cultured at 20 °C for 18 h to induce transformation. The grown cells were centrifuged for 20 min at 4000 rpm until harvesting. The cell pellet was re-suspended in 25 mL of 20 mM Tris-HCl (pH 7.0) and stored at − 80 °C.

To purify EZtag–Ag85B, the harvested cell lysate was thawed, 1 mM PMSF was added, and the cells were disrupted via pulse sonication. EZtag–Ag85B was purified from the resulting soluble fraction by precipitation and anion-exchange chromatography using a mono Q column. TEV protease was then added and allowed to react overnight at 30 °C. Pure Ag85B was obtained by removing the EZtag. Finally, the endotoxin in the purified Ag85B protein was removed using an endotoxin removal kit. The purified Ag85B protein was analyzed by sodium dodecyl sulfate-polyacrylamide gel electrophoresis (SDS-PAGE) on 10% gels.

### Preparation of Ag85B@aPNS

Ag85B-loaded aPNS (Ag85B@aPNS) was prepared via a simple nanoprecipitation method, which was slightly modified to prevent the formation of covalent bonds in proteins using a previously reported method [27]. First, amine-terminated PLA (10 mg) was completely dissolved in acetone (1 mL) and added to PF127 (20 mg). The mixture was allowed to react for 2 h. The resulting mixture was added dropwise to DIW (5 mL) under constant stirring and then placed in a fume hood overnight to remove acetone. After preparing the aPNS, various amounts of Ag85B (0, 0.1, 0.5, and 1 mg) were added to the aPNS solution and reacted at 4 °C under rotary shaking for 2 h. Finally, to purify Ag85B, which was not loaded into aPNS, Ag85B@aPNS was centrifuged using an Amicon Ultra-15 centrifugal filter unit with a molecular weight cut-off of 300 kDa.

The physicochemical characteristics of Ag85B@aPNS, namely the hydrodynamic diameter, hydrodynamic size distribution, polydispersity index (PDI), and zeta potential, were measured using a Zetasizer (ELS-Z2, Otsuka Electronics Co., Tokyo, Japan). The morphology of Ag85B@aPNS was observed using a transmission electron microscope (TEM; JEM-2100Plus HR, JEOL Co., Akishima, Tokyo, Japan). The Ag85B@aPNS suspension was dropped onto a TEM grid (Cu grid, 200 mesh) and air-dried in a desiccator for 72 h. Ag85B, which was not loaded into the aPNS, was assessed using micro-BCA to calculate the loading content and loading efficiency of Ag85B.

### Stability of Ag85B@aPNS

The colloidal stability of Ag85B@aPNS was investigated by lyophilization in a freeze-dryer (FDU-8606, Operon Co., Ltd., Gimpo, Republic of Korea) for 72 h. The stability of the Ag85B@aPNS lyophilizates was assessed by completely re-suspending the lyophilizates in DIW and subsequently comparing the physicochemical properties, such as the hydrodynamic diameter and PDI, of the Ag85B@aPNS solution before and after lyophilization [27].

The long-term stability of the Ag85B@aPNSs was examined in PBS, which mimics the physiological environment. Powdery Ag85B@aPNSs were immersed in PBS and maintained at 37 °C under gentle stirring. The characteristics of the Ag85B@aPNS solution were analyzed once every week for four weeks using a Zetasizer.

### In vitro release profile of Ag85B@aPNS

The release profile of Ag85B from Ag85B@aPNS was determined in accordance with a previously reported method [28]. Three dialysis devices (Float-A-Lyzer G2; molecular weight cut-off = 100 kDa) containing Ag85B@aPNS solution (1 mL) were established and subsequently placed in pre-warmed PBS (10 mL) in an incubator maintained at 37 °C under steady stirring (100 rpm). PBS containing released Ag85B from the dialysis device was collected at predetermined time points, and PBS in the dialysis device was replaced with fresh PBS. After 21 days, the amount of Ag85B in PBS collected at each time point was evaluated using a micro-BCA assay. The sample (25 µL) and mixture (200 µL) of micro-BCA reagents A and B were sufficiently reacted at 37 °C, and the color-changed samples were then examined at 562 nm using a microplate reader.

### In vitro cytotoxicity and cellular uptake

The cell viability assay was conducted using the MTT assay. Briefly, RAW 264.7 macrophages (50,000 cells/well) in a 96-well plate were treated with Ag85B and aPNS at increasing concentrations (0.05, 0.1, 4.1, 12.3, and 37.0 µg/mL) in a complete medium: RPMI-1640 medium supplemented with 10% FBS and 1% P/S. Before the assay, each well was washed with DPBS to remove extracellular materials and cell debris and treated with the MTT reagent. The plates were incubated in the dark at 37 °C for 3 h. Thereafter, 150 µL of MTT solvent was added to each well and left on a shaker in the dark for 15 min to dissolve the formazan crystals. Each well was pipetted to ensure adequate mixing, and the absorbance was measured at 590 nm using a Spectramax i3x (Molecular Devices, San Jose, CA, USA). Cell cytotoxicity data were calculated using the following equation:

$$\:Viability\:\left(\%\right)\hspace{0.17em}=\hspace{0.17em}[100\%\:\--\:cytotoxicity\:(\%\left)\right]$$

where cytotoxicity (%) = (absorbance value of the sample − absorbance value of untreated cells) × 100%.

To evaluate the cellular uptake capability of aPNS, a fluorescent-labeled model antigen (OVA-FITC) was loaded onto aPNS (OVA-FITC@aPNS). RAW 264.7 macrophages were seeded in 8-well chamber slides with 500,000 cells/well in 500 µL of DMEM and incubated for 12 h at 37°C in 5% CO_2_. Adherent cells were treated with 5 µg/mL of OVA-FITC and OVA-FITC@aPNS in a serum-free medium. After 6 h, the cells were washed twice with PBS and treated with 1 µM LysoTracker (Invitrogen, MA, USA) for 1 h at 37°C. For fixation, the cells were initially treated with a fixative solution (4% paraformaldehyde in PBS) for 10 min at 25°C. The cells were washed with PBS and then treated with 0.1% Triton™ X-100 (Sigma-Aldrich, St. Louis, USA). The cells were blocked in blocking buffer (1% bovine serum albumin (BSA) in PBS) for 30 min and counterstained with 4’,6-diamidino-2-phenylindole (DAPI; Invitrogen) for 30 min. The slides were washed, dried, and mounted for confocal fluorescence microscopy. Fluorescent images were obtained using a confocal laser scanning microscope (Carl Zeiss LSM 880, Baden-Württemberg, Germany), and images were acquired using the software ZEN 9.0 (Carl Zeiss).

### Animals and ethics statement

Four-to-five-week-old female C57BL/6 mice were purchased from Samtako (Seoul, Republic of Korea). The mice were maintained under standard environmental conditions with ad libitum access to commercial food and tap water (*ad libitum*). All mouse studies were performed in accordance with the guidelines provided by the Laboratory Animal Welfare and Ethics Committee, Korea Disease Control and Prevention Agency (KDCA), and in compliance with the Institutional Animal Care and Use Committee guidelines (Permit Number: KDCA-IACUC-22-035).

### Preparation of M. bovis BCG and M. tuberculosis cultures

BCG Pasteur 1173P2 and *M. tuberculosis* Erdman were provided by the KDCA and were grown in a medium containing Middlebrook’s 7H9 broth (Difco Laboratories, Detroit, MI, USA) supplemented with 10% oleic albumin dextrose catalase growth supplement (Becton Dickinson, Sparks, MD, USA) and 0.2% glycerol at 37 °C on a shaker at 100 rpm for 14–20 days. To prepare single-cell suspensions, mycobacterial cells were harvested via centrifugation at 10,000 *× g* for 20 min and washed three times with PBS (pH 7.2) [29]. The pellets were passed through 70-, 50-, 40-, 30-, 20-, 15-, and finally, 10-µm filters (Millipore Corp., MA, USA). The final stock was stored in small aliquots at − 80 °C until further use. Colony-forming units (CFUs) per milliliter of stock were measured using a counting assay on 7H10 agar plates.

### Animal immunization and aerosol infection

To assess the efficacy of the new TB vaccine, mice (5–6 weeks old; *n* = 5) were vaccinated with Ag85B@aPNS (aPNS encapsulates 25 µg of Ag85B) twice at an interval of three weeks via subcutaneous or intramuscular injections. One week after the final immunization (on day 28), mouse blood (*n* = 5) was collected to harvest sera for the measurement of antigen-specific IgG titers. Three weeks after the final immunization (after 6 weeks), the mice were challenged with the *M. tuberculosis* Erdman strain using a Glas-Col aerosol generator (Glas-Col LLC., IN, USA). The infection conditions were calibrated to expose each mouse to approximately 100 CFU [30].

To evaluate the immunogenicity of the BCG booster vaccine, mice (5–6 weeks old; *n* = 5) were subcutaneously, intramuscularly, and intranasally vaccinated (week 0) with BCG Pasteur 1173P2 (200,000 CFUs/mouse). This was followed by immunization with Ag85B@aPNS (aPNS-encapsulating 25 µg of Ag85B) twice at an interval of three weeks starting on day 42 (after 6 weeks) after BCG priming via subcutaneous injection. Three weeks after the final immunization (on day 84), the mice (*n* = 5) were euthanized by CO_2_ inhalation to analyze their immunogenicity. The immune response in mouse lung lymphocytes and splenocytes was assessed by measuring ex vivo responses using the ELISpot assay and IgG titer measurements.

### Measurement of serum IgG titer

Flat-bottom 96-well immunoplates (Thermo Fisher Scientific, MA, USA) were coated with 100 ng/mL Ag85B for 18 h at 4 °C. Serum samples were diluted to 1:200 using PBS containing 3% BSA and incubated for 2 h at 37 °C. The resulting samples were washed, and a 1:2,000 dilution of goat anti-mouse IgG-horseradish peroxidase (HRP) (Thermo Fisher Scientific) was added and incubated for 1 h at 37 °C. The substrate tetramethylbenzidine (TMB; Thermo Fisher Scientific) was added to each well, and the plate was incubated at 37 °C for 15–30 min. Thereafter, a stop solution for TMB was added, and the plates were read at 450 nm using a spectrophotometer (Spectramax i3x, Molecular Devices, CA, USA).

### CFU measurement and histopathology

To estimate the CFUs of *M. tuberculosis* in the lungs of infected mice, the lungs, except for the right superior lobe, were homogenized in 3 mL of PBS at 6 and 12 weeks after the challenge (*n* = 5 in each group). Next, 10-fold dilutions of the tissue homogenates were plated on 7H10 Middlebrook Agar (Difco Laboratories). The plates were incubated at 37 °C for 3–4 weeks, and the number of colonies was determined to assess the total CFUs in the lungs. For histopathological analysis, the right superior lobes of the lungs of infected mice were fixed in formalin (Sigma-Aldrich) and embedded in paraffin. Paraffin blocks were cut and stained with hematoxylin and eosin (H&E). A Motic Easy Scan scanner was used to scan the histological sections, and the images were analyzed to quantify granulomatous inflammation. The percentage of granulomatous tissue in the entire lung was calculated using ImageJ software (National Institutes of Health, Bethesda, Maryland, United States).

### Single-cell isolation

To evaluate the immunogenicity of the BCG booster vaccine, mouse lung lymphocytes, and splenocytes were isolated at the single-cell level. For single-cell preparation, the spleens and lungs were aseptically collected and pooled for each group. Spleens were homogenized for immune assays using a gentleMACS µTissue Dissociator (Miltenyl Biotec, Germany), washed in RPMI-1640 medium supplemented with 10% FBS and 1% P/S, rinsed in ammonium–chloride–potassium buffer to remove erythrocytes, and passed through a 40 μm cell strainer to generate single splenocytes. To isolate lymphocytes from mouse lungs, the tissue was incubated with DNase I (Roche, Basel, Switzerland) and collagenase D (Roche) in a plain medium at 37 °C for 1 h. The lymphocytes were separated from 5 to 20 mL of lung cell suspension on a Lymphoprep gradient (STEMCELL Technologies, Canada) via density centrifugation, passed through a 40 μm cell strainer, and re-suspended in RPMI-1640 medium containing 10% FBS and 1% P/S.

### ELISpot assay for IFN-γ secretion

The ELISpot assay was performed using an IFN-γ secretion ELISpot kit. Briefly, a single-cell suspension (1,000,000 cells) was stimulated with Ag85B and PPD (1 µg/mL) for 36 h at 37 °C in anti-IFN-γ antibody-coated filter plates. Thereafter, biotinylated anti-IFN-γ antibody, streptavidin-horseradish peroxidase conjugate, and 3-amino-9-ethylcarbazole were added as substrates to develop secreted cell spots, which were quantified using an Immunospot S6 analyzer (Cellular Immunospot Limited). The results are presented as the mean values of triplicate wells for each group.

### Quantification of cytokines

A single lung lymphocyte cell suspension (500,000 cells) was stimulated with Ag85B for 36 h at 37 °C, and the supernatant was collected to measure cytokine expression. The supernatants were diluted at a 1:2 ratio using a complete RPMI medium and assayed in triplicate wells for each group. The assays were performed using the Bio-Plex Pro Mouse Cytokine and Chemokine panel, which was customized to measure Interleukin-2 (IL-2), IL-12p(40), IL-17, monocyte chemoattractant protein-1 (MCP-1), macrophage inflammatory protein-1 alpha (MIP-1α) and beta (MIP-1β), keratinocyte-derived cytokines (KC), granulocyte/macrophage-colony stimulating factor (GM-CSF), and IL-1β (Bio-Rad Laboratories; Hercules, CA, USA), following the manufacturer’s instructions. The results were acquired using a Bio-Plex MAGPIX reader (Bio-Rad Laboratories), and the mean values were used to generate the graphs.

### Statistical analysis

All measurements were conducted in triplicate, and the results are presented as mean ± standard deviation. Statistical comparisons of animal experiments were performed using ordinary one-way ANOVA with Dunnett’s multiple comparisons test and are represented as the mean and SD. Significance were considered as follows: * *p* < 0.05, ** *p* < 0.01, *** *p* < 0.001, **** *p* < 0.0001.

## Results and discussion

### Preparation of Ag85B protein

The His-Ag85B plasmid was transformed and expressed in a bacterial host (*E. coli*) via IPTG induction. As shown in Figure S1(a), His-Ag85B was well expressed after IPTG induction. However, almost all His-Ag85B proteins formed insoluble aggregates. Therefore, Ag85B was cloned into a commercial EZtag fusion tag vector. The EZtag–Ag85B plasmid was transformed and expressed in a bacterial host (*E. coli*) via IPTG induction.

As shown in Figure S1 (b), unlike His-Ag85B, EZtag–Ag85B was well expressed and soluble. EZtag–Ag85B was purified via precipitation and anion-exchange chromatography. SDS-PAGE indicated that the purity of EZtag–Ag85B was 95%. Following cleavage with TEV protease, Ag85B with 97% purity was obtained (Figure S1 (c)and (d)).

### Physicochemical properties of Ag85B@aPNS

**Fig. 2:**
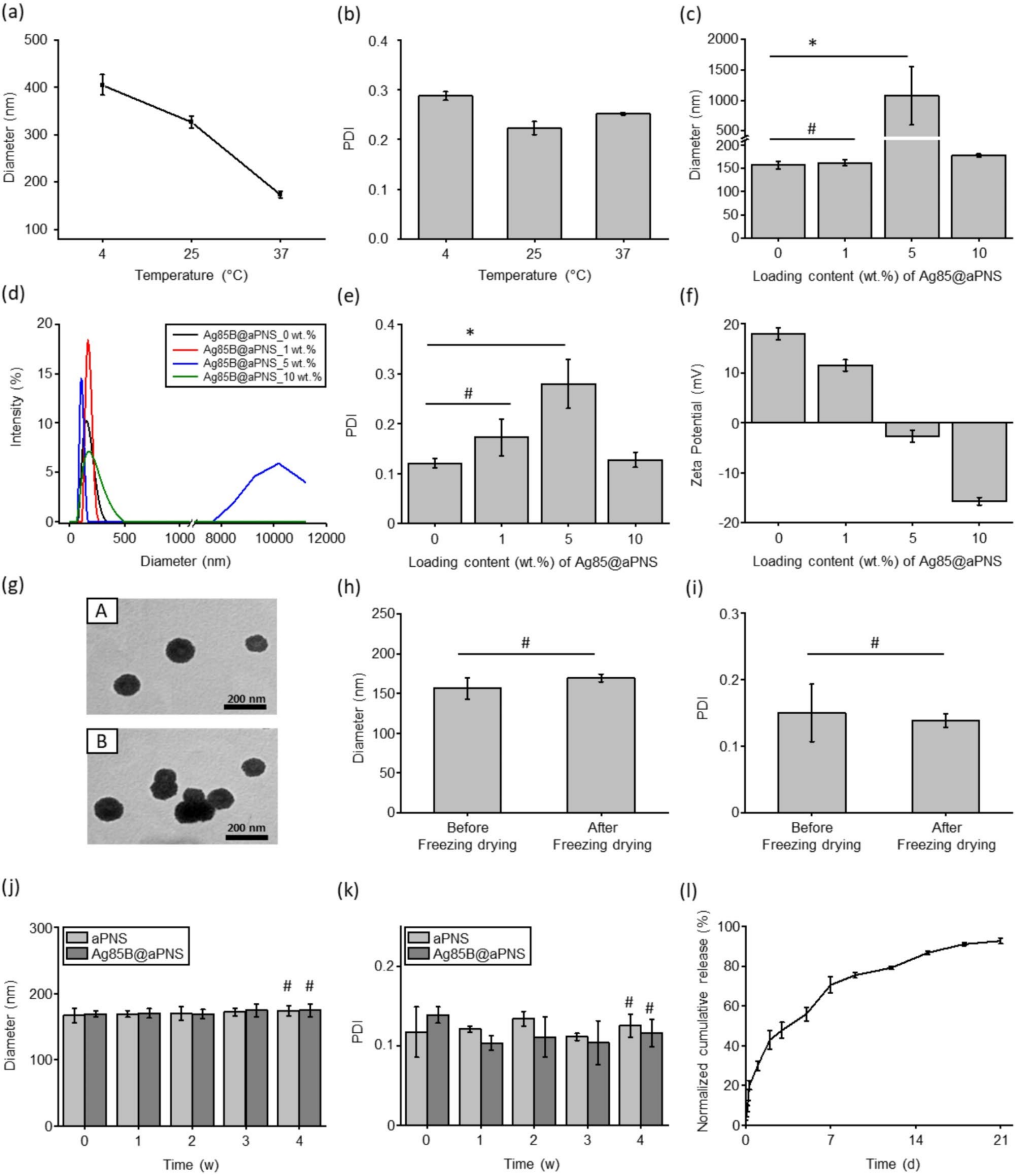
Physicochemical characteristics of Ag85B@aPNS. (**a**, **b**) Temperature-dependent swelling properties of the aPNS. (**c**) Hydrodynamic diameter, (**d**) size distribution, (**e**) polydispersity index (PDI), and (**F**) zeta potential of Ag85B@aPNS with varying Ag85B loadings. (**g**) Representative morphological images of aPNS (**A**) and Ag85B@aPNS (**B**); scale bars = 200 nm. Stability of Ag85B@aPNS: (**h**) Hydrodynamic diameter and (**i**) PDI of Ag85B@aPNS re-suspended in deionized water (DIW) after freeze-drying. (**j**) Hydrodynamic diameter and (**k**) PDI of aPNS and Ag85B@aPNS in PBS at 37 °C. Values were analyzed using Student’s t-test and one-way ANOVA; #: *p* > 0.05 and *: *p* < 0.05. (l) Release profile of Ag85B from Ag85B@aPNS in PBS, evaluated using micro-BCA (mean ± SD, *n* = 3)

Temperature-responsive aPNS were successfully prepared by simple nanoprecipitation without any covalent chemistry. The prepared aPNS consisted of an amine-terminated PLA core and a Pluronic shell with a core-to-shell ratio of 1:2. According to a previous report [31], Pluronic displayed similar lower critical solution temperature (LCST) behaviors over wide temperature ranges; whereas it deswells above its LCST, it swells below it. These phenomena are caused by entropy resulting from hydrophobic interactions between the hydrophobic (PPO) and hydrophilic (PEO) segments of the polymer chains [32]. To confirm the temperature-responsive behavior of aPNS, we examined its temperature dependence. As shown in Fig. 2(a) and (b), the prepared aPNS exhibited temperature-responsive properties such as changes in size and PDI: ~400 nm and 0.288 at 4 °C, ~ 320 nm and 0.223 at 25 °C, and ~ 170 nm and 0.252 at 37 °C. The volume expansion at low temperatures can be ascribed to the destruction of the aPNS structure by the hydrophobic interactions of the shell. In addition, this behavior was reversible when thermally cycled at 4–37 °C. As shown in Figure S2, the aPNS did not show significant changes in diameter after successive thermal cycles. These results demonstrate that the aPNS maintained structural stability during repeated swelling–deswelling.

Based on the above-mentioned characteristics of aPNS, we loaded Ag85B into aPNS by relying on the temperature-dependent swelling property of Pluronic. Furthermore, Ag85B was efficiently loaded inside the aPNS via electrostatic interactions between the positively charged aPNS and negatively charged Ag85B. The physicochemical characteristics of Ag85B@aPNSs prepared with various amounts of Ag85B were evaluated. As shown in Fig. 2(c), compared to aPNS, there was an increase in the size of Ag85B@aPNS with over 5 wt% Ag85B was statistically significant (* *p* < 0.05), whereas the change in the hydrodynamic diameter (almost 160 nm) up to 1 wt% loading of Ag85B was not statistically significant. As shown in Fig. 2(d) and (e), Ag85B@aPNS with ~ 1 wt% Ag85B showed mono-phase, narrow hydrodynamic diameter distribution, and PDI below 0.3. In contrast, Ag85B@aPNS with 5 wt% Ag85B displayed bi-phase distribution and a PDI ranging from 0.24 to 0.32, and Ag85B@aPNS with 10 wt% Ag85B showed a broad size distribution despite being mono-phase with a PDI below 0.3. In particular, the change in the zeta potential up to 1 wt% Ag85B was negligible, and the zeta potentials were in the range of 13–15 mV (Fig. 2(f)). However, when the amount of negatively charged Ag85B encapsulated into aPNS increased, the zeta potential changed gradually from − 2 mV (Ag85@aPNS with 5 wt%) to − 15 mV (Ag85B@aPNS with 10 wt%). The morphological changes in the aPNS before and after encapsulation with 1 wt% Ag85B were examined via TEM, and the results are shown in Fig. 2(g). The size and morphology of the aPNS (Fig. 2(g)-A) were similar to those of Ag85B@aPNS with 1 wt% Ag85B (Fig. 2(g)-B). Additionally, their spherical morphologies, assessed using a Zetasizer, were comparable. These findings indicate that the 1 wt% Ag85B is the optimal loading amount for preparing Ag85B@aPNS without significant changes in the physicochemical and morphological characteristics of aPNS.

### Stability and drug release behavior

Colloidal stability is an important factor in the development of drug-delivery nanoparticle systems because the instability of nanoparticles can physically affect uncontrolled release behavior or particle aggregation [33]. Although lyophilization can promote stability, cryoprotectants, stabilizers, and surfactants are required to inhibit stress from crystal formation, ice-water interactions, and phase separation during long-term storage [33, 34]. Unlike most nanoparticles, Ag85B@aPNS comprises Pluronic as an FDA-approved stabilizer. Therefore, during the experiments, the stability of Ag85B@aPNS was determined via lyophilization without the addition of cryoprotectants or surfactants. To examine the stability of Ag85B@aPNS after lyophilization, changes in the size and PDI of re-suspended Ag85B@aPNS were determined. As shown in Fig. 2**(h)** and **(i)**, the hydrodynamic diameter and PDI of lyophilized Ag85B@aPNS were not significantly different from those of Ag85B@aPNS before lyophilization.

Furthermore, to investigate the functioning of the nanoparticles under complex biological conditions, aPNS and Ag85B@aPNS were monitored in a physiological environment for four weeks. During the experimental period, they showed sufficient stability, and the changes in hydrodynamic diameter and PDI were not statistically significant (Fig. 2**(j)** and **(k)**). These observations demonstrate that Ag85B@aPNS can be lyophilized without additional cryoprotectants, re-suspended without significant changes in physicochemical properties, and is stable for a long period in environments that mimic biological conditions. Good colloidal stability is advantageous for achieving long-term pharmaceutical stability during vaccination [35].

The sustained-release behavior of Ag85B from aPNS in PBS at 37 °C was examined for 21 days, as shown in Fig. 2**(l)**. The amount of released Ag85B reached 30, 70, and 93% on days 1, 7, and 21, respectively. Thus, the amount of Ag85B required to maintain therapeutic efficacy can be prolonged by using aPNS without an initial burst.

### In vitro cell viability and phagocytosis of Ag85B@aPNS

**Fig. 3:**
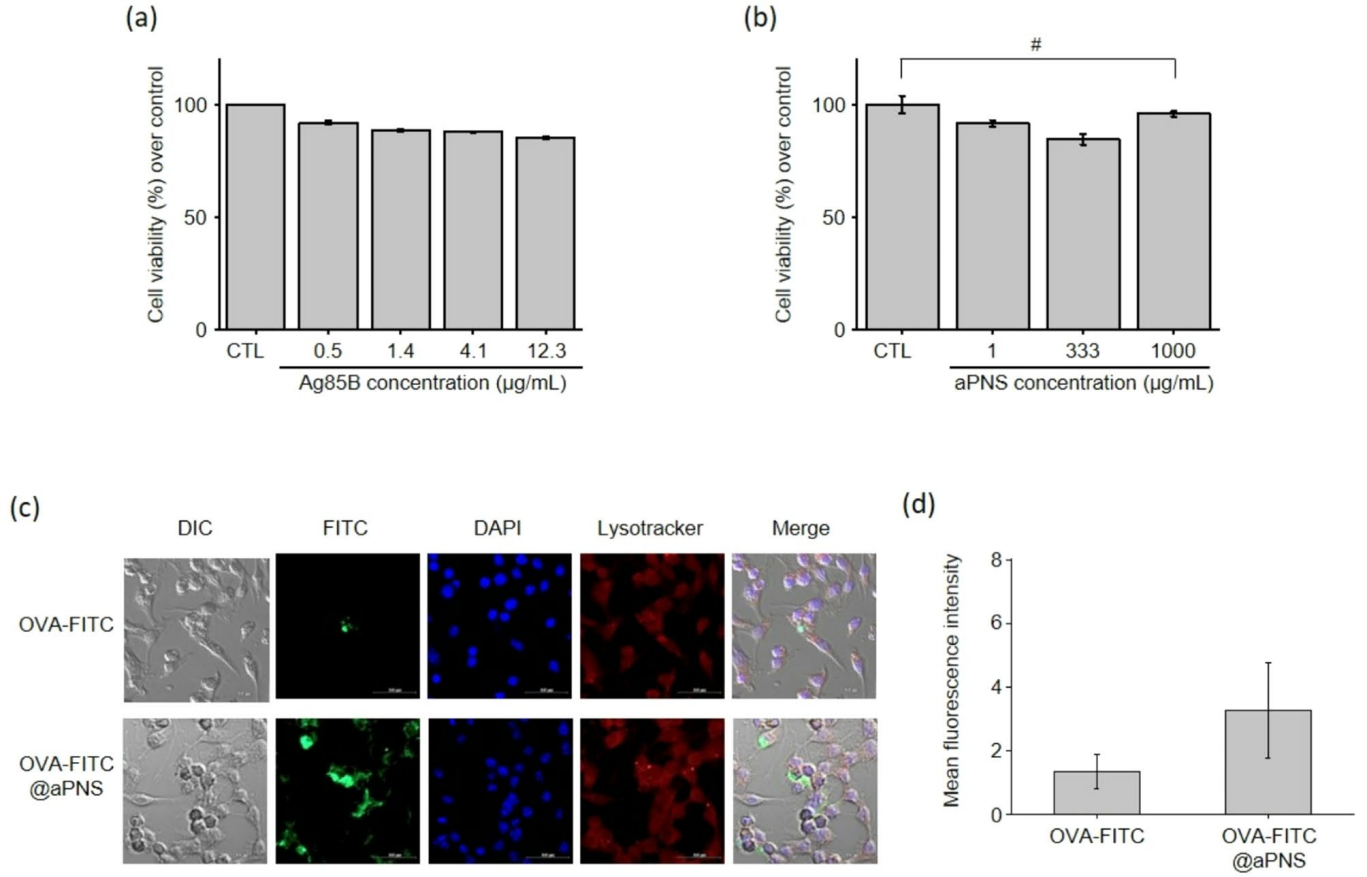
In vitro cytotoxicity and phagocytosis in macrophages. (**a**) Cytotoxicity of Ag85B protein and (**b**) aPNS at various concentrations was assessed using an MTT assay in RAW 264.7 macrophages. Phagocytosis of aPNS by antigen-presenting cells: (**a**) Representative confocal images of OVA-FITC and OVA-FITC@aPNS phagocytosed by RAW 264.7 macrophages, showing OVA-FITC (green), nuclei stained with DAPI (blue), and LysoTracker (red); scale bar = 100 μm. (**b**) Quantification of cellular phagocytosis as mean fluorescence intensity relative to unphagocytosed cells

Cytotoxicity is a key parameter for evaluating the biocompatibility of nanoparticles. The physicochemical characteristics of nanoparticles, such as particle size, can contribute to potential cytotoxicity. Smaller nanoparticles can easily interact with cellular components—such as proteins, carbohydrates, nucleic acids, and fatty acids—potentially inducing cellular damage by entering the cells [36]. Therefore, we investigated the cytotoxicity of Ag85B and aPNS in macrophages. First, we examined the cytotoxicity of the synthesized and purified Ag85B. RAW 264.7 macrophages were treated with Ag85B at concentrations ranging from 0.5 to 12.3 µg/mL. As shown in Fig. 3(a), Ag85B concentrations below 12.3 µg/mL exhibited no cytotoxic effects, with cell viability exceeding 85%. Subsequently, we assessed the cytotoxicity of aPNS at concentrations up to 1 mg/mL. As shown in Fig. 3(b), aPNS showed no significant cytotoxicity or statistical differences within this concentration range. These results suggest that both Ag85B (at concentrations below 12.3 µg/mL) and aPNS (at concentrations below 1 mg/mL) do not affect cell viability; thus, the non-cytotoxic concentrations of Ag85B and aPNS were established for further cellular uptake experiments.

Additionally, examining macrophage surface receptors engaged in nanoparticle detection is crucial for understanding the adjuvanticity of aPNS. Phagocytosis of nanoparticles by antigen-presenting cells triggers immunological and inflammatory processes following vaccine immunization [37]. The cellular uptake capacity of aPNS was investigated using confocal laser scanning microscopy with aPNS-encapsulating FITC-labeled OVA. OVA-FITC@aPNS exhibited noticeably more intense green fluorescence in the macrophage nuclei than the OVA-FITC-treated group (Fig. 3(c)). In addition, the fluorescence intensity of OVA-FITC@aPNS was approximately 2.5 times higher than that of free OVA-FITC (Fig. 3(d)). Moreover, OVA-FITC@aPNS-treated cells showed lysosomal distribution, which is a process of phagolysosomes. These observations confirmed that aPNS were effectively phagocytized by macrophages without inducing any cytotoxicity.

### Protective effectiveness of Ag85B@aPNS in *M. tuberculosis*-infected mice

**Fig. 4:**
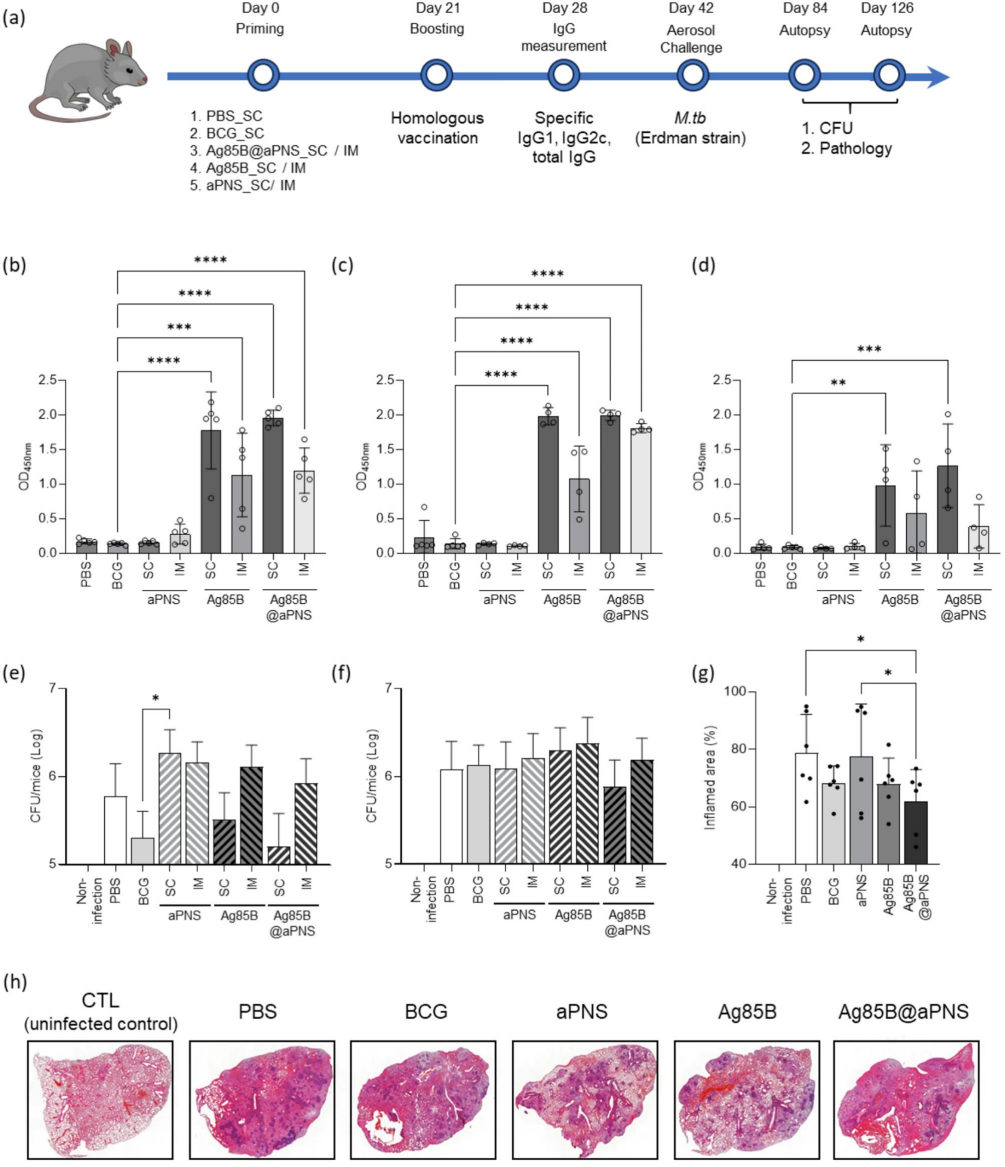
Protective effectiveness of Ag85B@aPNS in *M. tuberculosis*-infected mice. (**a**) Schematic representation of immunization schedule and further evaluation. The mice were divided into BCG-only, aPNS-only, Ag85B-only, and Ag85B@aPNS groups. The aPNS, Ag85B, and Ag85B@aPNS groups were further divided into two groups depending on the route of immunization: subcutaneous (SC) and intramuscular (IM). After 4 weeks (day 28), mouse blood was collected, and sera were isolated to measure Ag85B-specific (**b**) total IgG, (**c**) IgG1, and (**d**) IgG2c. Twelve weeks after the last immunization (day 84), the vaccinated mice were challenged with the Erdman strain via the aerosol route (*n* = 5 per group). The mice were euthanized, and their lungs were harvested after infection periods of (**e**) 6 and (**F**) 15 weeks to enumerate bacilli. The right superior lung lobe of each mouse was collected. (**g**) The inflamed area was stained using H&E staining, and the percentage of inflamed area in the overall lung lesions was calculated. Values were analyzed using One-way ANOVA with Dunnett’s multiple comparisons test and are represented as the mean and SD. Significance was considered as follows: ** *p* < 0.01, *** *p* < 0.001, and **** *p* < 0.0001

To investigate the potential of Ag85B@aPNS as a novel tuberculosis (TB) vaccine, a prime-boost vaccination strategy was implemented. C57BL/6 mice (*n* = 5 per group) were subcutaneously immunized with PBS or BCG and either subcutaneously or intramuscularly immunized with aPNS, Ag85B, or Ag85B@aPNS. Immunizations were administered twice at three-week intervals, except for BCG, and antigen-specific antibody titers were measured three weeks after the final boost, prior to the challenge (Fig. 4(a)). Previous studies have indicated that the neutralizing antibody response against immunodominant pathogen epitopes may contribute to protection against *M. tuberculosis* through growth restriction within the lung cells [38, 39]. Therefore, the levels of Ag85B-specific antibodies (IgG, IgG1, and IgG2c) were measured in the serum of each group of mice. IgG subclasses represent T helper cell subsets related to the immune response. We examined IgG1 and IgG2c, which facilitate T helper type 2 (Th2) and 1 (Th1) responses, respectively. As shown in Fig. 4(b)–(d), the level of antibody response was regulated according to the administration route and formulation. Unlike the aPNS-immunized group that showed no difference in immune responses compared to PBS and BCG, a noteworthy difference in the levels of IgG, IgG1, and IgG2c was observed for the Ag85B and Ag85B@aPNS groups. Remarkably, Ag85B@aPNS-immunized mice treated with a subcutaneous regimen generated higher Ag85B-specific and IgG2c titers than the other groups, implying that Ag85B@aPNS facilitates a Th1 type response, which is crucial for host defense against *M. tuberculosis* [40].

Subsequently, to examine the protective effectiveness of Ag85B@aPNS, C57BL/6 mice were aerosol-challenged with *M. tuberculosis*, and the bacterial loads in the lung after 6 and 15 weeks were recorded. As shown in Fig. 4(e)–(g), compared with the PBS group, the TB-vaccinated mice (BCG, Ag85B, and Ag85B@aPNS groups) showed a reduction in the bacterial lung load. Notably, among the various formulations with different administration routes, subcutaneously immunized Ag85B@aPNS mice demonstrated significantly higher efficacy, which was maintained until 15 weeks post-infection. To further support these findings, the lungs of subcutaneously vaccinated mice were stained with H&E to investigate inflammatory regions. Ag85B@aPNS-immunized mice showed the greatest reduction in the frequency of inflamed areas in autopsied lung tissue at 15 weeks post-infection (Fig. 4(h), Figure S3). These results demonstrate that Ag85B@aPNS has a protective effect via the Th1 response, highlighting the potential of Ag85B@aPNS as a novel TB vaccine.

### Immune response induced by Ag85B@aPNS in a BCG-primed mouse

**Fig. 5:**
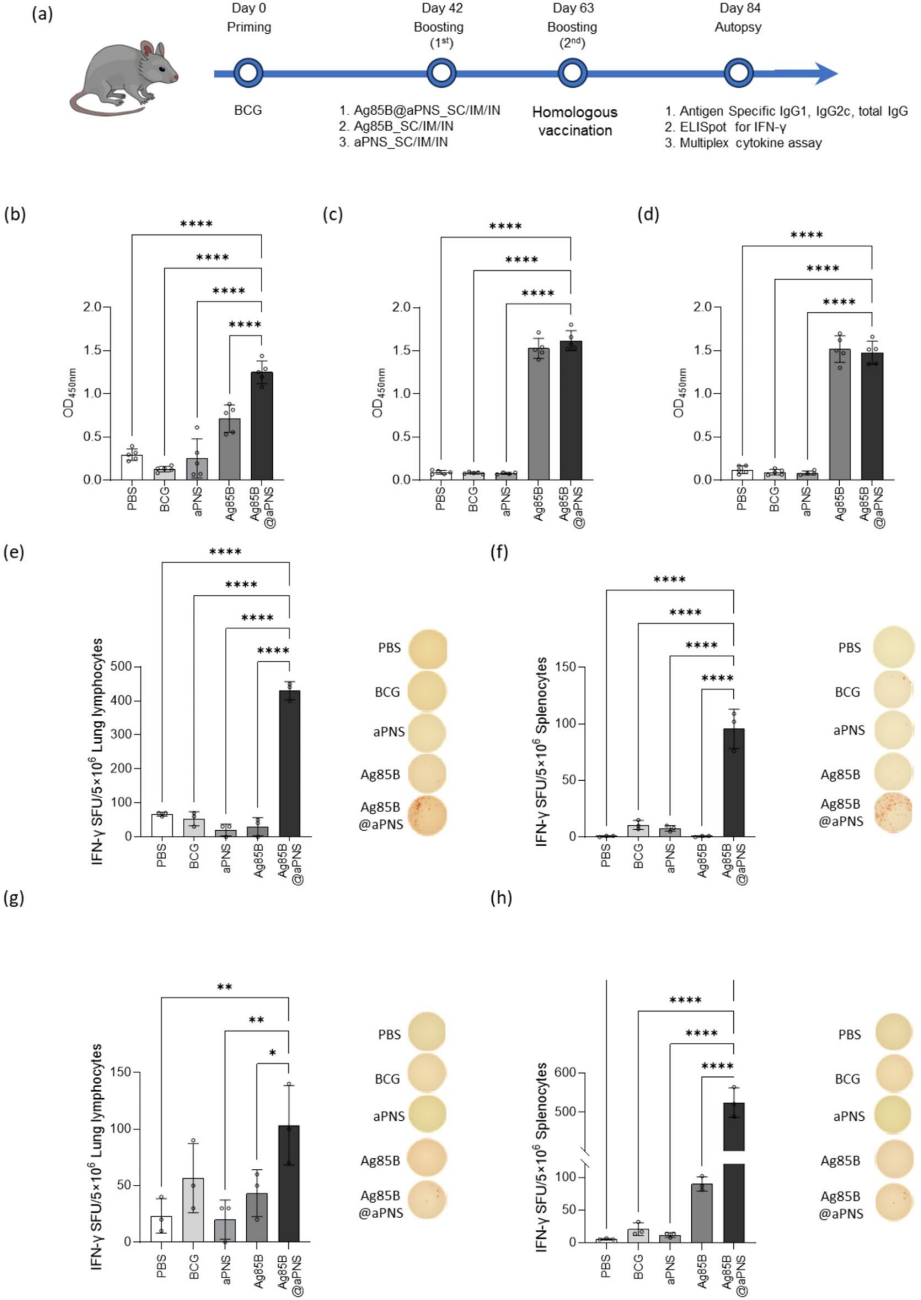
Immunogenicity of Ag85B@aPNS as a BCG-booster vaccine. (**a**) Schematic of the immunization schedule and further evaluation as a BCG-booster vaccine. Mice were divided into BCG-only, BCG-primed aPNS, BCG-primed Ag85B, and BCG-primed Ag85B@aPNS-immunized groups. Three weeks after the final immunization, the mice were euthanized, and blood, lung lymphocytes, and splenocytes were collected. Ag85B-specific (**b**) total IgG, (**c**) IgG1, and (**d**) IgG2c levels were measured in sera isolated from mice. IFN-γ secreted by single cells was detected using the ELISpot assay after 36 h of incubation with (**e**, **F**) Ag85B protein (100 ng/mL) and (**g**, **h**) PPD (100 ng/mL). Data are shown as lung lymphocytes (**e** and **g**) and splenocytes (**F** and **h**), accompanied by representative photographs of spot-forming cells. Values were analyzed using One-way ANOVA with Dunnett’s multiple comparisons test and are represented as the mean and SD. Significance was considered as follows: * *p* < 0.05, ** *p* < 0.01, **** *p* < 0.0001

Encouraged by the results presented above, we hypothesized that Ag85B@aPNS could also play the role of a BCG booster vaccine. Therefore, we investigated the ability of Ag85B@aPNS to boost the immune response induced by BCG administration. The mice were subcutaneously immunized with BCG. Thereafter, they were subcutaneously immunized with Ag85B@aPNS, Ag85B, or aPNS twice at three-week intervals via the SC, IM, and intranasal (IN) routes. On day 84, humoral and cell-mediated immune responses were assessed (Figure S3). In the lung lymphocytes of the respective groups, immunogenicity following BCG and aPNS immunization was comparable to that of the PBS group. However, the Ag85B@aPNS-immunized group exhibited a significantly higher induction of antigen-specific T-cell responses in SC immunization. Based on these results, we confirmed the effectiveness of this approach for subsequent experiments involving SC immunization, following an identical animal experiment schedule (Fig. 5(a)). The levels of Ag85B-specific IgG, IgG1, and IgG2c were measured, and the responses following BCG and aPNS immunization were similar to those observed in the PBS group. However, compared to BCG, the levels of Ag85B-specific IgG, IgG1, and IgG2c in the Ag85B@aPNS immunized group were high and statistically significant. Although the levels of IgG1 and IgG2c in Ag85B@aPNS- and Ag85B-immunized mice were similar, total IgG levels were augmented by Ag85B@aPNS immunization (Fig. 5(b)–(d)). To determine the number of IFN-γ-secreted T cells, ELISpot assays of mouse splenocytes and lung lymphocytes were conducted. The number of IFN-γ spot forming units (SFU) was determined after re-stimulation with Ag85B and PPD. As shown in Fig. 5(e)–(h), cytokine expression in the splenocytes and lung lymphocytes of mice immunized with Ag85B@aPNS was significantly higher than that in the BCG-, Ag85B-, and aPNS-immunized groups re-stimulated with both Ag85B and PPD. This result implies that the vaccine candidate proposed herein, Ag85B@aPNS, could boost the immune response induced by BCG administration.

**Fig. 6:**
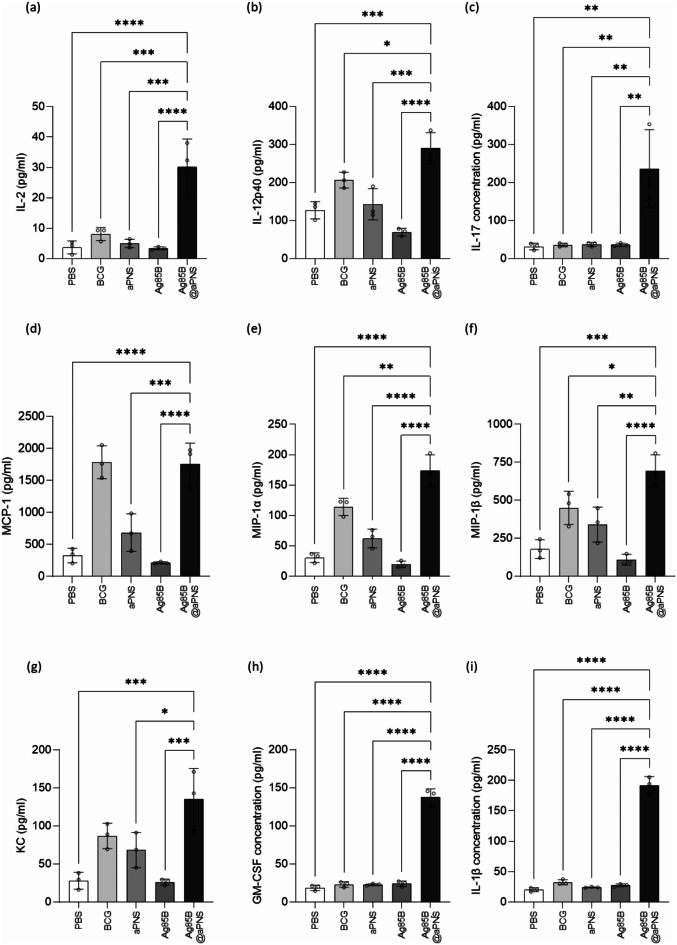
Cytokine production is related to Th1 and macrophage activation. Lung lymphocytes were harvested from euthanized mice. Cultured supernatants of lung lymphocytes were collected for cytokine level measurement after stimulation with Ag85B protein (100 ng/mL) for 36 h at 37 °C. The production levels of six cytokines and chemokines: (**a**) IL-2, (**b**) IL-12p40, (**c**) IL-17, (**d**) MCP-1, (**e**) MIP-1α, (**F**) MIP-1β, (**g**) KC, (**h**) GM-CSF, and (**i**) IL-1β were examined using a bead-based multiplex cytokine assay. Values were analyzed using One-way ANOVA with Dunnett’s multiple comparisons test and are represented as the mean and SD. Significance were considered as follows: *: *p* < 0.05, **: *p* < 0.01, ***: *p* < 0.001, ****: *p* < 0.0001

In addition, we investigated the vaccine-induced immune response by measuring the levels of pro-inflammatory cytokines and chemokines, including IL-2, IL-12p40, IL-17, MCP-1, MIP-1α, MIP-1β, KC, GM-CSF, and IL-1β, in the supernatants of cultured lung lymphocytes (Fig. 6). IL-2 and IL-12p40, which are integral to Th1 cell responses, as well as IL-17, a crucial mediator of protective immunity via Th17 cell responses [41], were significantly elevated in the Ag85B@aPNS group compared to the other groups (Fig. 6(a)–(c). Furthermore, the Ag85B@aPNS group exhibited increased levels of MCP-1, MIP-1α, and MIP-1β, which play critical roles in recruiting inflammatory cells and sustaining effector immune responses related to pathogenesis (Fig. 6(d)–(f)) [42, 43]. Moreover, the administration of Ag85B@aPNS resulted in elevated levels of cytokines and chemokines associated with the activity of innate immune cells. Specifically, KC (also known as CXCL-1), which is produced by peritoneal macrophages in response to TLR signaling to recruit neutrophils during pathogen invasion [44], as well as GM-CSF and IL-1β, which modulate the functionality of innate immune cells [45, 46], were significantly increased following Ag85B@aPNS administration (Fig. 6(g)–(i)). These results suggest that Ag85B@aPNS enhances immune responses by activating innate immune cells, such as macrophages, thereby linking Th1 and Th17 cell responses.

## Conclusion

In this study, we designed Ag85B@aPNS as a novel TB- and BCG booster vaccine. Ag85B was successfully expressed and purified with sufficient yield using a fusion tag. The successfully assembled Ag85B was encapsulated via electrostatic interactions and swelling of aPNS. The aPNS favorably loaded with 1 wt% Ag85B without leading to a change in its physicochemical properties. In addition, Ag85B@aPNS exhibited good stability after lyophilization in the absence of cryoprotectants under long-term physiological conditions. In vitro experiments showed that aPNS and Ag85B did not exhibit notable cytotoxicity and that aPNS boosted the phagocytic capability of Ag85B. Furthermore, Ag85@aPNS administered via subcutaneously stimulated an enhanced Th1 and Th17 immune response, resulting in a considerable reduction in the bacterial load in the lungs and pulmonary inflammatory lesions in *M. tuberculosis-*challenged mice. Furthermore, BCG-primed mice immunized with Ag85B@aPNS showed improved humoral and cell-mediated immune responses. These observations suggest that Ag85B@aPNS could be used as an alternative to BCG as well as a BCG booster vaccine candidate against TB.

## Electronic supplementary material

Below is the link to the electronic supplementary material.

Supplementary Material 1

## Data Availability

“Data will be made available on request” was included in the manuscript.

## Abbreviations

BSA: Bovine Serum Albumin
CFU: Colony-Forming Units
DPBS: Dulbecco’s Phosphate-Buffered Saline
FBS: Fetal Bovine Serum
H&E: Hematoxylin and Eosin
KC: Keratinocyte-derived Cytokines
KDCA: Korea Disease Control and Prevention Agency
KFRM: Korean Fund for Regenerative Medicine
LCST: Lower Critical Solution Temperature
PBS: Phosphate-Buffered Saline
SFU: Spot Forming Units
TEM: Transmission Electron Microscope
TEV: Tobacco Etch Virus

## References

[1] Liang Z, Li M, Ni J, Hussain T, Yao J, Song Y, et al. CFP10-loaded PLGA nanoparticles as a booster vaccine confer protective immunity against Mycobacterium bovis. Bioimpacts. 2022;12:395–404. 10.34172/bi.2022.23645. 36381632 10.34172/bi.2022.23645 PMC9596879

[2] Chopra H, Mohanta YK, Rauta PR, Ahmed R, Mahanta S, Mishra PK, et al. An In-sight into advances in developing Nanotechnology based therapeutics, Drug Delivery, Diagnostics and vaccines: multidimensional applications in tuberculosis dis-ease management. Pharmaceuticals (Basel). 2023;16:581. 10.3390/ph16040581. 37111338 10.3390/ph16040581 PMC10145450

[3] Dadu A, Yedilbayev A, Migliori GB, Ahmedov S, Falzon D, den Boon S, et al. PASS to end TB in Europe: accelerated efforts on prevention and systematic screening to end tuberculosis in the WHO European Region by 2030. Int J Infect Dis. 2024;141S:106980. 10.1016/j.ijid.2024.02.023. 38403111 10.1016/j.ijid.2024.02.023

[4] Dijkman K, Lindenstrom T, Rosenkrands I, Soe R, Woodworth JS, Lindestam Arlehamn CS, et al. A protective, single-visit TB vaccination regimen by co-administration of a subunit vaccine with BCG. NPJ Vaccines. 2023;8:66. 10.1038/s41541-023-00666-2. 37160970 10.1038/s41541-023-00666-2 PMC10169149

[5] World Health Organization, Global tuberculosis report. 2023. https://www.who.int/teams/global-tuberculosis-programme/tb-reports/global-tuberculosis-report-2023. Access date 15 January 2025.

[6] Pierneef L, van Hooij A, de Jong D, Tjon Kon Fat EM, van Meijgaarden KE, Petruccioli E, et al. Host biomarker-based quantitative rapid tests for detection and treatment monitoring of tuberculosis and COVID-19. iScience. 2023;26:105873. 10.1016/j.isci.2022.105873. 36590898 10.1016/j.isci.2022.105873 PMC9791715

[7] Kuan R, Muskat K, Peters B, Lindestam Arlehamn CS. Is mapping the BCG vaccine-induced immune responses the key to improving the efficacy against tuberculosis? J Intern Med. 2020;288:651–60. 10.1111/joim.13191. 33210407 10.1111/joim.13191 PMC9432460

[8] Wang J, Fan XY, Hu Z. Immune correlates of protection as a game changer in Tuberculosis vaccine development. NPJ Vaccines. 2024;9:208. 10.1038/s41541-024-01004-w. 39478007 10.1038/s41541-024-01004-w PMC11526030

[9] van Meijgaarden KE, Li W, Moorlag S, Koeken V, Koenen H, Joosten LAB, et al. BCG vaccination-induced acquired control of mycobacterial growth differs from growth control preexisting to BCG vaccination. Nat Commun. 2024;15:114. 10.1038/s41467-023-44252-5. 38167829 10.1038/s41467-023-44252-5 PMC10761850

[10] Zhuang L, Ye Z, Li L, Yang L, Gong W, Next-Generation TB. Vaccines: Progress, challenges, and prospects. Vaccines (Basel). 2023;11. 10.3390/vaccines11081304. 10.3390/vaccines11081304 PMC10457792 37631874

[11] Gyu Choi H, Woong Kwon K, Jae Shin S. Importance of adjuvant selection in Tuberculosis vaccine development: exploring basic mechanisms and clinical implications. Vaccine X. 2023;15:100400. 10.1016/j.jvacx.2023.100400. 37965276 10.1016/j.jvacx.2023.100400 PMC10641539

[12] Veerapandian R, Gadad SS, Jagannath C, Dhandayuthapani S. Live attenuated vaccines against tuberculosis: targeting the disruption of genes encoding the Secretory Proteins of Mycobacteria. Vaccines (Basel). 2024;12. 10.3390/vaccines12050530. 10.3390/vaccines12050530 PMC11126151 38793781

[13] Komine-Aizawa S, Mizuno S, Matsuo K, Namiki T, Hayakawa S, Honda M. Recombinant BCG-Prime and DNA-Boost immunization confers mice with enhanced protection against Mycobacterium kansasii. Vaccines (Basel). 2021;9. 10.3390/vaccines9111260. 10.3390/vaccines9111260 PMC8618695 34835191

[14] Aghababa H, Mobarez AM, Behmanesh M, Khoramabadi N, Mobarhan M. Production and purification of Mycolyl Transferase B of Mycobacterium tuberculosis. Tanaffos. 2011;10:23–30. 25191384 PMC4153168

[15] Dang S, Li W, Wen S, Song Y, Bai M, Li S, et al. Ag85a-S2 activates cGAS-STING signaling pathway in intestinal mucosal cells. Vaccines (Basel). 2022;10. 10.3390/vaccines10122170. 10.3390/vaccines10122170 PMC9785823 36560581

[16] Singh S, Saavedra-Avila NA, Tiwari S, Porcelli SA. A century of BCG vaccination: Immune mechanisms, animal models, non-traditional routes and implications for COVID-19. Front Immunol. 2022;13:959656. 10.3389/fimmu.2022.959656. 36091032 10.3389/fimmu.2022.959656 PMC9459386

[17] Guthrie CM, Tan X, Meeker AC, Self AE, Liu L, Cheng Y. Engineering a dual vaccine against COVID-19 and Tuberculosis. Front Cell Infect Microbiol. 2023;13:1273019. 10.3389/fcimb.2023.1273019. 37965265 10.3389/fcimb.2023.1273019 PMC10641007

[18] Liu X, Min Q, Song H, Yue A, Li Q, Zhou Q, et al. Potentiating humoral and cellular immunity using a novel hybrid polymer-lipid nanoparticle adjuvant for HBsAg-VLP vaccine. J Nanobiotechnol. 2023;21:441. 10.1186/s12951-023-02116-6. 10.1186/s12951-023-02116-6 PMC10666313 37993870

[19] Enriquez AB, Izzo A, Miller SM, Stewart EL, Mahon RN, Frank DJ, et al. Advancing adjuvants for Mycobacterium tuberculosis therapeutics. Front Immunol. 2021;12:740117. 10.3389/fimmu.2021.740117. 34759923 10.3389/fimmu.2021.740117 PMC8572789

[20] Zhao T, Cai Y, Jiang Y, He X, Wei Y, Yu Y, et al. Vaccine adjuvants: mechanisms and platforms. Signal Transduct Target Ther. 2023;8:283. 10.1038/s41392-023-01557-7. 37468460 10.1038/s41392-023-01557-7 PMC10356842

[21] Alqahtani MS, Kazi M, Ahmad MZ, Syed R, Alsenaidy MA, Albraiki SA. Lignin nanoparticles as a promising vaccine adjuvant and delivery system for ovalbumin. Int J Biol Macromol. 2020;163:1314–22. 10.1016/j.ijbiomac.2020.07.026. 32645499 10.1016/j.ijbiomac.2020.07.026

[22] Nooraei S, Sarkar Lotfabadi A, Akbarzadehmoallemkolaei M, Rezaei N. Immunogenicity of different types of adjuvants and Nano-adjuvants in Veterinary vaccines: a Comprehensive Review. Vaccines (Basel). 2023;11. 10.3390/vaccines11020453. 10.3390/vaccines11020453 PMC9962389 36851331

[23] Lee BM, Park SJ, Noh I, Kim CH. The effects of the molecular weights of hyaluronic acid on the immune responses. Biomater Res. 2021;25:27. 10.1186/s40824-021-00228-4. 34462017 10.1186/s40824-021-00228-4 PMC8404285

[24] Dong J, Wang W, Zhou W, Zhang S, Li M, Li N, et al. Immunomodulatory biomaterials for implant-associated infections: from conventional to advanced therapeutic strategies. Biomater Res. 2022;26:72. 10.1186/s40824-022-00326-x. 36471454 10.1186/s40824-022-00326-x PMC9721013

[25] Ballester M, Nembrini C, Dhar N, de Titta A, de Piano C, Pasquier M, et al. Nanoparticle conjugation and pulmonary delivery enhance the protective efficacy of Ag85B and CpG against tuberculosis. Vaccine. 2011;29:6959–66. 10.1016/j.vaccine.2011.07.039. 21787826 10.1016/j.vaccine.2011.07.039

[26] Malik A, Gupta M, Mani R, Bhatnagar R. Single-dose Ag85B-ESAT6-loaded poly(lactic-co-glycolic acid) nanoparticles confer protective immunity against tuberculosis. Int J Nanomed. 2019;14:3129–43. 10.2147/IJN.S172391. 10.2147/IJN.S172391 PMC6501725 31118627

[27] Lee JS, Hwang Y, Oh H, Sung D, Tae G, Choi WI. All-in-one nanosponge with pluronic shell for synergistic anticancer therapy through effectively overcoming multidrug resistance in cancer. Nanomedicine. 2022;40:102486. 10.1016/j.nano.2021.102486. 34748960 10.1016/j.nano.2021.102486

[28] Flores-Valdez MA, Velazquez-Fernandez JB, Pedroza-Roldan C, Aceves-Sanchez MJ, Gutierrez-Ortega A, Lopez-Romero W, et al. Proteome and immunogenicity differences in BCG Pasteur ATCC 35734 and its derivative, the vaccine candidate BCGDeltaBCG1419c during planktonic growth in 7H9 and Proskauer Beck media. Tuberculosis (Edinb). 2024;144:102432. 10.1016/j.tube.2023.102432. 38041962 10.1016/j.tube.2023.102432

[29] Cole ST, Brosch R, Parkhill J, Garnier T, Churcher C, Harris D, et al. Deciphering the biology of Mycobacterium tuberculosis from the complete genome sequence. Nature. 1998;393:537–44. 10.1038/31159. 9634230 10.1038/31159

[30] Kwon KW, Lee A, Larsen SE, Baldwin SL, Coler RN, Reed SG, et al. Long-term protective efficacy with a BCG-prime ID93/GLA-SE boost regimen against the hyper-virulent Mycobacterium tuberculosis strain K in a mouse model. Sci Rep. 2019;9:15560. 10.1038/s41598-019-52146-0. 31664157 10.1038/s41598-019-52146-0 PMC6820558

[31] Choi SH, Lee JH, Choi SM, Park TG. Thermally reversible pluronic/heparin nanocapsules exhibiting 1000-fold volume transition. Langmuir. 2006;22:1758–62. 10.1021/la052549n. 16460102 10.1021/la052549n

[32] Ramos J, Forcada J, Hidalgo-Alvarez R. Cationic polymer nanoparticles and nanogels: from synthesis to biotechnological applications. Chem Rev. 2014;114:367–428. 10.1021/cr3002643. 24003911 10.1021/cr3002643

[33] Trenkenschuh E, Friess W. Freeze-drying of nanoparticles: how to overcome colloidal instability by formulation and process optimization. Eur J Pharm Biopharm. 2021;165:345–60. 10.1016/j.ejpb.2021.05.024. 34052428 10.1016/j.ejpb.2021.05.024

[34] Mahmud MM, Pandey N, Winkles JA, Woodworth GF, Kim AJ. Toward the scale-up production of polymeric nanotherapeutics for cancer clinical trials. Nano Today. 2024;56. 10.1016/j.nantod.2024.102314. 10.1016/j.nantod.2024.102314 PMC11155436 38854931

[35] Muramatsu H, Lam K, Bajusz C, Laczko D, Kariko K, Schreiner P, et al. Lyophilization provides long-term stability for a lipid nanoparticle-formulated, nucleoside-modified mRNA vaccine. Mol Ther. 2022;30:1941–51. 10.1016/j.ymthe.2022.02.001. 35131437 10.1016/j.ymthe.2022.02.001 PMC8815268

[36] Heyns IM, Faunce AF, Mumba MN, Kumar M, Arora M. Nanotechnology-enhanced naloxone and alternative treatments for opioid addiction. ACS Pharmacol Transl Sci. 2024;7:2237–50. 10.1021/acsptsci.4c00158. 39144549 10.1021/acsptsci.4c00158 PMC11320732

[37] Back PI, Yu M, Modaresahmadi S, Hajimirzaei S, Zhang Q, Islam MR, et al. Immune implications of cholesterol-containing lipid nanoparticles. ACS Nano. 2024;18:28480–501. 10.1021/acsnano.4c06369. 39388645 10.1021/acsnano.4c06369 PMC11505898

[38] Li H, Javid B. Antibodies and tuberculosis: finally coming of age? Nat Rev Immunol. 2018;18:591–96. 10.1038/s41577-018-0028-0. 29872140 10.1038/s41577-018-0028-0

[39] Achkar JM, Casadevall A. Antibody-mediated immunity against tuberculosis: implications for vaccine development. Cell Host Microbe. 2013;13:250–62. 10.1016/j.chom.2013.02.009. 23498951 10.1016/j.chom.2013.02.009 PMC3759397

[40] Wangoo A, Sparer T, Brown IN, Snewin VA, Janssen R, Thole J, et al. Contribution of Th1 and Th2 cells to protection and pathology in experimental models of granulomatous lung disease. J Immunol. 2001;166:3432–9. 10.4049/jimmunol.166.5.3432. 11207301 10.4049/jimmunol.166.5.3432

[41] Khader SA, Bell GK, Pearl JE, Fountain JJ, Rangel-Moreno J, Cilley GE, et al. IL-23 and IL-17 in the establishment of protective pulmonary CD4 + T cell responses after vaccination and during Mycobacterium tuberculosis challenge. Nat Immunol. 2007;8:369–77. 10.1038/ni1449. 17351619 10.1038/ni1449

[42] Singh S, Anshita D, Ravichandiran V. MCP-1: function, regulation, and involvement in disease. Int Immunopharmacol. 2021;101:107598. 10.1016/j.intimp.2021.107598. 34233864 10.1016/j.intimp.2021.107598 PMC8135227

[43] Olszewski MA, Huffnagle GB, McDonald RA, Lindell DM, Moore BB, Cook DN, et al. The role of macrophage inflammatory protein-1 alpha/CCL3 in regulation of T cell-mediated immunity to Cryptococcus neoformans infection. J Immunol. 2000;165:6429–36. 10.4049/jimmunol.165.11.6429. 11086082 10.4049/jimmunol.165.11.6429

[44] De Filippo K, Henderson RB, Laschinger M, Hogg N. Neutrophil chemokines KC and macrophage-inflammatory protein-2 are newly synthesized by tissue macrophages using distinct TLR signaling pathways. J Immunol. 2008;180:4308–15. 10.4049/jimmunol.180.6.4308. 18322244 10.4049/jimmunol.180.6.4308

[45] Benmerzoug S, Marinho FV, Rose S, Mackowiak C, Gosset D, Sedda D, et al. GM-CSF targeted immunomodulation affects host response to M. Tuberculosis infection. Sci Rep. 2018;8:8652. 10.1038/s41598-018-26984-3. 29872095 10.1038/s41598-018-26984-3 PMC5988704

[46] Silverio D, Goncalves R, Appelberg R, Saraiva M. Advances on the role and applications of Interleukin-1 in tuberculosis. mBio. 2021;12:e0313421. 10.1128/mBio.03134-21. 34809460 10.1128/mBio.03134-21 PMC8609357

