# Supplementary material for: A temperature-responsive PLA-based nanosponge as a novel nanoadjuvant and efficient delivery carrier of Ag85B for effective vaccine against Mycobacterium tuberculosis
Source: Cell Commun Signal. 2025 Apr 1;23:159. doi: 10.1186/s12964-025-02105-2 (PMC11963517; doi:10.1186/s12964-025-02105-2)
Supplement: Supplementary file 1 — Supplementary Material 1 [file 12964_2025_2105_MOESM1_ESM.docx]

**A temperature-responsive PLA-based nanosponge as a novel nanoadjuvant and efficient delivery carrier of Ag85B for effective vaccine against *Mycobacterium tuberculosis***

Jin-Seung Yun^a,b,#^, Soo-Min Kim^a,c,#^, Jin Sil Lee^d,e,#^, Su Hyun Jeong^d,f^, Hyeryeon Oh^d,g^, Panmo Son^d,h^, Sunghyun Kim^d^, Young-Ran Lee^d^, Eunkyung Shin^a^, Sang-Jun Ha^b^, Yong-Woo Jung^c^, Dokeun Kim^a^, Hye-Sook Jeong^a*^, and Won Il Choi^d*^

*^a^ National Institute of Infectious Disease, Korea National Institute of Health, 212, Osongsaengmyeong 2-ro, Osong-eup, Heungdeok-gu, Cheongju, Chungbuk 28160, Republic of Korea*

*^b^ Department of Biochemistry, College of Life Science and Biotechnology, Yonsei University, Seoul 03722, Republic of Korea*

*^e^ College of Pharmacy, Korea University, Sejong 30019, Republic of Korea*

*^d^ Bio-Convergence Materials R&D Division, Korea Institute of Ceramic Engineering and Technology, 202, Osongsaengmyeong 1-ro, Osong-eup, Heungdeok-gu, Cheongju, Chungbuk 28160, Republic of Korea*

*^e^ Drug Manufacturing Center, Daegu-Gyeongbuk Medical Innovation Foundation (K-MEDI Hub), Daegu, 41061, Republic of Korea*

*^f^ Department Of Bioengineering， Hanyang University，Seoul 04763, Republic of Korea*

*^g^ School of Materials Science and Engineering and Department of Nanobio Materials and Electronics, Gwangju Institute of Science and Technology, 261 Cheomdan-gwagiro, Buk-gu, Gwangju 500-712, Republic of Korea*

*^h^ Department of Applied Bioengineering, Graduate School of Convergence Science and Technology, Seoul National University, Seoul 08826, Republic of Korea*

^#^ These authors contributed equally to this work.

^*^ Corresponding authors;

E-mail: choi830509@kicet.re.kr; tel.: +82-43-913-1513; fax: +82-43-913-1597 (Won Il Choi)

E-mail: jeongnih@korea.kr; tel.: +82-43-913-4300; fax: +82-43-913-8189 (Hye-Sook Jeong)


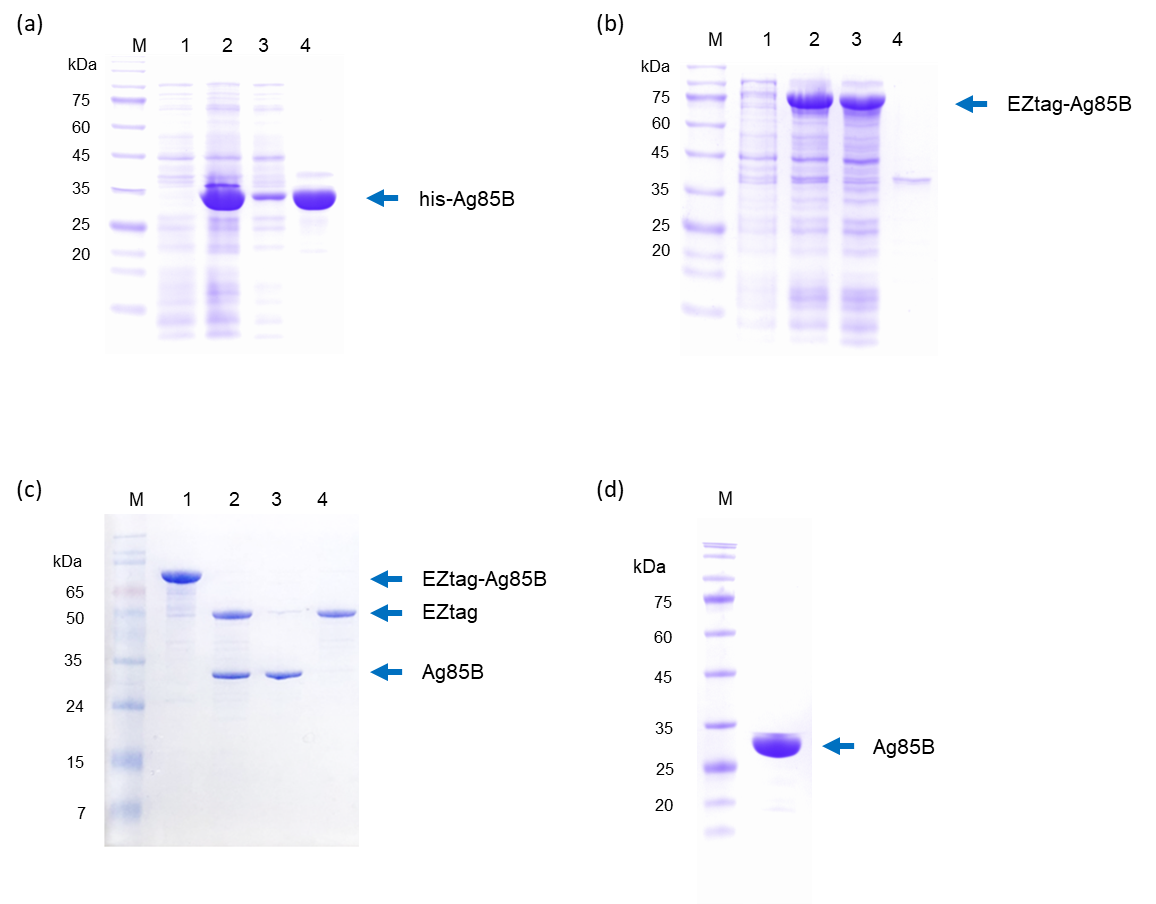


**Figure S1.** SDS-PAGE analysis of the Ag85B purification steps produced in *E. coli* BL21(DE3) strain. (a) SDS-PAGE analysis of the expression and purification steps for His-Ag85B. (M: Marker; lane 1: before IPTG induction; lane 2: after IPTG induction; lane 3: supernatant of cell lysate; lane 4: pellet of cell lysate.) (b) SDS-PAGE analysis of the expression and purification steps for EZtag-Ag85B. (M: Marker; lane 1: before IPTG induction; lane 2: after IPTG induction; lane 3: supernatant of cell lysate; lane 4: pellet of cell lysate.) (c) SDS-PAGE analysis of the Ag85B purification steps. (M: Marker; lane 1: purified EZtag-Ag85B after anion exchange chromatography; lane 2: after cleavage with TEV protease of EZtag-Ag85B; lane 3: purified Ag85B after anion exchange chromatography; lane 4: separated EZtag after anion exchange chromatography.) (d) SDS-PAGE analysis of purified Ag85B.

**
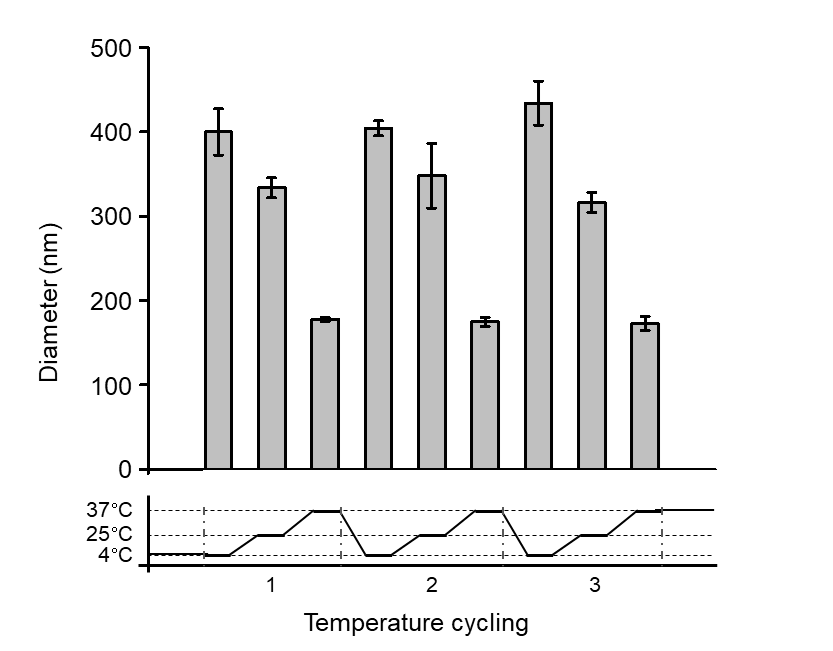
**

**Figure S2**. Temperature-induced reversible swelling and deswelling of aPNS were observed at 4 °C, 25 °C, and 37 °C during temperature cycling.


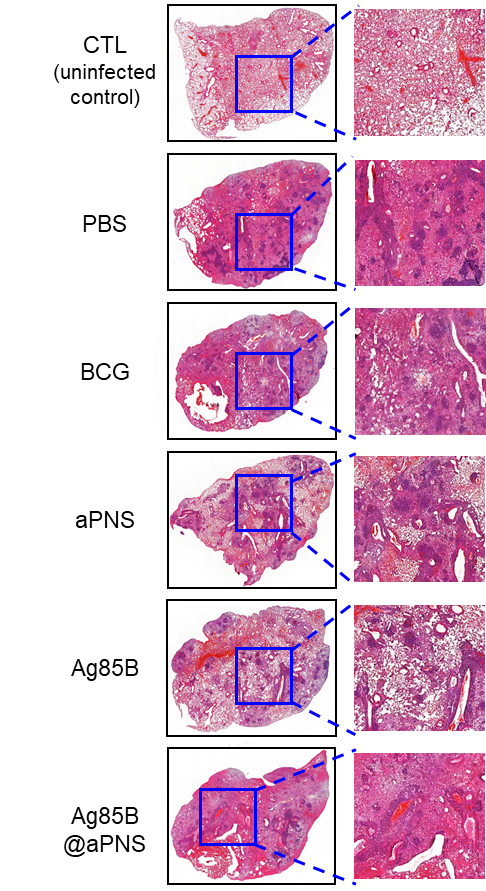


**Figure S3**. Magnified characteristic lesions in pathological images of lung tissues from *M. tuberculosis*-infected mice.

**Figure S4**. Immunogenicity of Ag85B@aPNS as a BCG-booster vaccine, evaluated across subcutaneous (SC), intramuscular (IM), and intranasal (IN) immunization routes.
